# Digital Patient-Reported Outcome Measures for Monitoring of Patients on Cancer Treatment: Cross-sectional Questionnaire Study

**DOI:** 10.2196/18502

**Published:** 2021-08-13

**Authors:** Mayuran Ananth Sivanandan, Catherine Sharma, Pippa Bullard, Judith Christian

**Affiliations:** 1 Department of Oncology and Radiotherapy, Nottingham University Hospitals NHS Trust Nottingham United Kingdom

**Keywords:** patient-reported outcome measures, patient-reported outcomes, remote monitoring, toxicity, outpatients, digital technology, digital health, mobile health, oncology, chemotherapy, immunotherapy, radiotherapy

## Abstract

**Background:**

Oncology has been facing increasing outpatient activity associated with higher cancer incidence, better survival rates, and more treatment options. Innovative technological solutions could help deal with this increasing demand. Using digital patient-reported outcome measures (PROMs) to identify patients who need a face-to-face (FTF) appointment is a potential approach.

**Objective:**

This study aims to assess the feasibility of digital PROM questionnaires to enable remote symptom monitoring for patients undergoing cancer treatment and their ability to highlight the requirement for an FTF appointment.

**Methods:**

This study was performed at a tertiary oncology center between December 2018 and February 2019. The Common Terminology Criteria for Adverse Events were adapted into patient-friendly language to form the basis of treatment-specific digital questionnaires covering specific cancer drugs and radiotherapy treatments. These treatment-specific digital PROM questionnaires were scored by both patients and their clinicians during FTF appointments. Patients and clinicians did not see each other’s scored PROMs. Agreement between patients and clinicians was assessed using descriptive statistics. Patient and staff feedback was also obtained.

**Results:**

In total, 90 patients participated in the study across 10 different treatment pathways. By comparing paired patient and clinician responses, the sensitivity of the patient-completed questionnaires in correctly highlighting the need for FTF review was 94% (44/47), and all patients with severe or grade 3+ symptoms were identified (6/6, 100%). Patient-completed PROMs appropriately revealed that 29% (26/90) of the participating patients did not need FTF review based on their symptoms alone. Certain oncological treatment pathways, such as immunotherapy, were found to have a larger proportion of patients with minimal symptoms than others, such as conventional chemotherapy. Patient and staff feedback showed high approval of digital PROMs and their potential for use in remote monitoring.

**Conclusions:**

Digital PROM questionnaires can feasibly highlight the need for FTF review in oncology clinics for treatment. Their use with specific treatments could safely reduce the requirement for FTF care, and future work should evaluate their application in the remote monitoring of patients.

## Introduction

### Background

Oncology is a predominantly outpatient specialty; hence, the increases in outpatient activity are of particular relevance. There has been an increase in National Health Service (NHS) outpatient appointments in England from 63.2 million to 118.6 million in the 10-year period ending between 2016 and 2017 [1] and projected significant increases in the demand for oncology services in both the United States [2] and Europe [3]. Growing service pressures on oncology outpatient activities are specifically driven by increased cancer incidence [4], improved survival rates [5], and an expanded treatment repertoire [6]. Current pressures on outpatient services have been stated to negatively affect patient and clinician experience [7]. Furthermore, global workforce shortages are increasing and are predicted to increase further [2,3,8]. Therefore, the outpatient system will struggle to continue to offer the capacity to deal with the increasing demand in its current traditional form.

Consequently, improving the efficiency of oncology care is paramount [2]; for example, the UK NHS’ Long Term Plan advocates a fundamental remodeling of outpatients working with technology to help drive a reduction in face-to-face (FTF) outpatient appointments of up to a third in the coming 5 years [9]. This is particularly relevant given the impact of the COVID-19 pandemic. An application of technology that will help achieve this ambitious target is to allow alternative consultation methods outside a traditional FTF encounter to review patients. An example is remote monitoring, where technology can allow patients’ health to be checked at a distance by clinical staff, such as through the completion of symptom-related questionnaires incorporating patient-reported outcome measures (PROMs) [7,10].

The clinical benefits of PROMs being used as a part of the care of patients with cancer have been shown to include increased awareness of symptoms by patients and clinicians, streamlining of consultations, improved interprofessional communication [11], and improved health care outcomes for patients, including quality of life and survival [12]. Furthermore, their use is associated with patient-centered care and improved patient self-efficacy [13]. The use of PROMs and digital technology has been advocated in cancer strategy reports by the NHS [14] and the Independent Cancer Taskforce [15].

The strategy of using PROMs in monitoring patients remotely has been applied successfully in gastroenterology in patients with inflammatory bowel disease on immunosuppressive treatment [16]. A similar strategy would be equally attractive in oncology, where a large proportion of follow-up activities involve regular attendance to monitor patients on treatments [17], including both radiotherapy (RT) and systemic treatments. In the research setting, the use of electronic PROMs to allow regular reporting of chemotherapy side effects by patients on cancer treatment has been evaluated in the context of randomized controlled trials. These studies have indicated several improved patient outcomes, such as improved quality of life and reduced hospitalization rates through improved symptom management [12,18-20]. However, the data for actually replacing routine FTF outpatient follow-up of patients on oncological treatment with remote monitoring with PROMs in the standard setting are sparse [21].

For patients on cancer treatments, the National Cancer Institute Common Terminology Criteria for Adverse Events (CTCAE) [22] is the standard tool used by clinicians to grade and record treatment-related adverse events (Textbox 1), and this is typically done in *on treatment* outpatient clinics. Many adverse events are based on a patient’s subjective experience, and this has led to individual groups rephrasing CTCAE, which are designed for clinicians, into a patient-understandable language to generate a PROM that directly captures the patient perspective and maintains the clinical usefulness of CTCAE [23-25]. The National Cancer Institute has developed its own PROM based on CTCAE (patient-reported outcomes Common Terminology Criteria for Adverse Events [PRO-CTCAE]) for use in patients in cancer clinical trials [26]; however, it does not currently map onto the severity grades of CTCAE that are used for clinical decision-making.

**Common Terminology Criteria for Adverse Events grading for adverse events.**

**Common Terminology Criteria for Adverse Events (CTCAE) Grade and Description Adapted From National Cancer Institute (2017): CTCAE (Version 5.0) [22]**

- Grade 0: no symptom
- Grade 1: mild symptom not needing intervention
- Grade 2: moderate symptom where intervention is indicated
- Grade 3: severe symptom that requires hospitalization

Additional benefits of using a PROM in gauging oncological treatment–related adverse events are that evidence suggests that clinicians can underreport their severity [27] and that the recording of toxicity by clinicians in routine practice can be suboptimal [28]. Trials that have evaluated the utility of patient-modified CTCAE as a PROM have examined its use in addition to existing FTF hospital appointments [12,25,29], and it has not been assessed as a tool to help determine whether an FTF appointment is actually needed.

Our oncology department represents the largest oncological facility in the East Midlands [30] in one of the largest hospital trusts in England [31]. Locally, the department’s outpatient activity has consistently increased on an annual basis, with a growth of approximately 2500 appointments per year on average over the last 6 years. A major driver for this increase was found to be *on treatment* appointments. Therefore, strategies to reduce footfall within the oncology outpatient department would be beneficial.

### Objective

In this context, it was hypothesized that remotely completed questionnaires based on patient-modified CTCAE could serve as a triage tool to ascertain the need for a patient to attend an FTF appointment. It was believed that PRO-CTCAE would not be appropriate in this setting as it does not map onto the CTCAE severity grades that are clinically relevant to help determine the need for an FTF appointment; moreover, PRO-CTCAE is advocated to be used only for symptoms that occurred in the previous 7 days [26]. We, therefore, performed a feasibility study at our center to assess if digital PROM questionnaires based on patient-modified CTCAE could be used for symptom monitoring in oncology *on treatment* clinics and compared paired patient and clinician questionnaires to identify whether these questionnaires could accurately highlight the requirement for an FTF appointment.

## Methods

### Overview

A cross-sectional study was undertaken at Nottingham University Hospitals NHS Trust (United Kingdom) to evaluate the use of digital PROM questionnaires between December 2018 and February 2019. A multi-professional team led this project, and input was sought from clinicians, information technology staff, quality improvement specialists, and patient representatives. The technology partner for this project was DrDoctor (London), who provided an electronic portal to allow the completion of questionnaires.

### Patient Groups

Specific oncological treatment pathways for this feasibility study were chosen to cover the breadth of both radical RT and systemic drug pathways. For the systemic drug pathways, the study included several patient groups who were considered less likely to have significant side effects on treatment, such as single-agent immunotherapy patients and patients on oral targeted drugs. A similar theme was chosen for the RT group; therefore, patients with adjuvant breast and radical prostate RT were targeted. Nevertheless, it was also decided to test in some groups, such as metastatic prostate cancer patients on chemotherapy and patients with radical RT for head and neck cancer, where the opportunities for FTF reduction in care might be less obvious.

### PROM Development

It was decided that treatment-specific PROMs would be designed to assess treatment-related symptoms and side effects. The symptoms that needed assessment, and therefore, inclusion in each treatment-specific questionnaire, were decided by a project-team clinician through review of the appropriate treatment-specific literature (eg, summary of product characteristics) and trusted UK cancer information websites [32]. Subsequently, appropriate questions were developed by adapting relevant items of the CTCAE [22] and World Health Organization Performance Status (PS) for relevant questionnaires pertaining to systemic treatment, into a patient-friendly language in a similar approach to previous groups [25,27,29]. Responses to CTCAE items were based on grades on an ordinal scale of 0 (not present), 1 (mild), 2 (moderate), and 3 (severe) and PS on a scale from 0 to 4.

It was recognized that certain symptoms, such as fever or palpitations, were more appropriate for a binary question (yes-no) alone rather than an ordinal-scale question, and this approach was used where required. It was also deemed that the questionnaires should determine the presence of emotional concerns in patients. There was no appropriate CTCAE item to capture this; therefore, a binary question about emotional concerns was created by clinicians and added to all questionnaires. A collaborative approach with site-specific oncological teams was implemented with a review of relevant treatment questionnaires before use. They made comments and suggested amendments that were enacted before the questionnaires were used by patients in this study.

### Digital Interface Development

**Figure 1:**
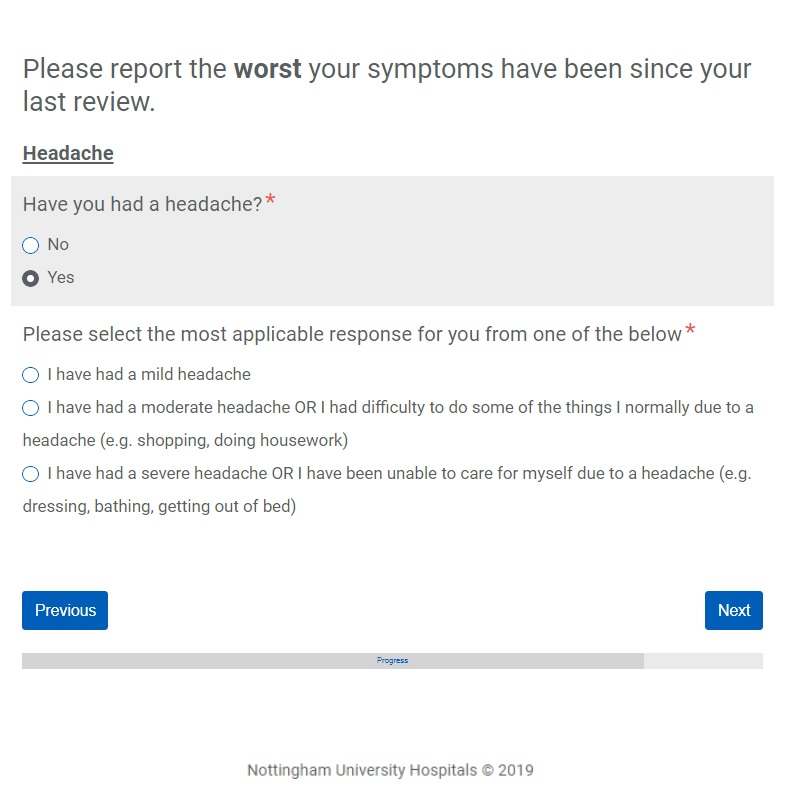
An example of a question from a digital patient-reported outcome measure questionnaire.

The questionnaires were converted into a digital format by a member of the information technology team using the internet-based Formstack system (Formstack LLC) and subsequently uploaded to the DrDoctor portal, which is a cloud-based platform. The design of the electronic questionnaires was based on the work of previous research groups whose electronic questionnaire design was found to be acceptable to patients [18,29]. Apart from the PS question, symptom occurrence had to be indicated by answering a yes-no question, and if yes was selected, the corresponding graded responses would appear for a patient to mark as appropriate. The authors felt this would minimize the amount of reading for patients and thus the burden on their time. Each question had to be answered before moving to the next to ensure that all questions were completed. Questionnaires were designed to be simple to reduce break-off rates [33], and a progress bar was placed at the bottom of each page to increase the likelihood of completing the survey [34]. An example of a question from a digital PROM questionnaire is shown in Figure 1.

The DrDoctor portal is password-protected, and a member of the study team allocated the appropriate treatment-specific questionnaires for patients and clinicians to complete during the study. Completed questionnaires contained no patient-identifiable data and were assigned a letter to allow corresponding patient and clinician questionnaires to be analyzed for concordance.

### Study Design

Patients eligible for this study were recommended by their clinical teams; they had to be aged at least 18 years, able to understand written English, and have specific cancer diagnoses currently receiving specific treatments (Table 1). Patients had to provide verbal informed consent, and patients unable to complete the questionnaires were excluded from the study. Eligible patients were approached by a member of the study team to complete a treatment-specific digital PROM questionnaire in the oncology outpatient department and RT review clinics at Nottingham University Hospitals NHS Trust. Patients completed the digital PROM questionnaire before their FTF appointment unless the time pressures of their FTF appointment required completion after their FTF appointment. This was deemed acceptable, as a previous study showed no significant difference if patients completed their questionnaires before or after seeing their clinician [27]. The patients completed the questionnaire electronically on a tablet device in a private room in the outpatient department with a member of the study team. Clinicians were asked to complete a corresponding digital PROM questionnaire following a participating patient’s FTF appointment. CTCAE was common knowledge to all clinicians before this study, but comprehensive knowledge of the precise CTCAE symptom grades was not required as questionnaire responses were designed to equate to the appropriate CTCAE grading. The process of asking both patients and clinicians to score symptoms blind of each other was a new process needed for this study.

**Table 1:** Patient characteristics (N=90).

Treatment pathway	Tumor site	Treatment intent	Patients, n (%)
Capecitabine chemotherapy with or without oxaliplatin chemotherapy	Colorectal	Adjuvant	11 (12)
Abiraterone and enzalutamide	Prostate	Palliative	10 (11)
Breast radiotherapy	Breast	Adjuvant	10 (11)
Head and neck radiotherapy	Head and neck	Radical	10 (11)
Single-agent immunotherapy (nivolumab or pembrolizumab)	Melanoma	Palliative and adjuvant	10 (11)
Prostate radiotherapy	Prostate	Radical	10 (11)
Pazopanib	Renal	Palliative	10 (11)
Imatinib	Gastrointestinal stromal tumor	Palliative and adjuvant	8 (9)
Stereotactic ablative radiotherapy	Lung	Radical	6 (7)
Docetaxel chemotherapy	Prostate	Palliative	5 (6)

The rationale for clinicians completing a corresponding electronic questionnaire was to enable the comparison of paired responses between patients and clinicians. The current standard outpatient pathway for the assessment of treatment-related side effects is dependent on a clinician’s interpretation of a patient’s symptoms; therefore, comparison of paired patient and clinician questionnaires would enable assessment of the feasibility and accuracy of a patient-completed PROM on its own to triage the need for further assessment. Patients and clinicians did not see each other’s PROM results, and the results were not used for clinical decision-making. This was done so that the suitability of our designed questionnaires could be assessed before consideration for routine clinical use. The current standard of care for FTF appointments was maintained for all patients to ensure patient safety. Our method is similar to that of other groups who have performed similar interventions [25,27].

Participants were asked to complete a feedback form enquiring about the usability of the digital questionnaire, thoroughness of the questionnaire, and acceptance of future use on a 10-point Likert scale. Participating study clinicians were asked to complete a similar feedback form after the completion of the study. The authors wanted to assess not only the user experience with the digital interface but also the content of the questionnaires. Hence, the authors designed a bespoke feedback form to assess both because it was not possible to use a pre-existing tool, such as the System Usability Scale, which is solely focused on usability. It was decided that the feedback form would comprise 3 questions to maximize response rates. A 10-point Likert scale was chosen to enable sufficient distinction between positive and negative responses and generate quantitative data for analysis [35]. Examples of feedback form questions can be found in Multimedia Appendices 1 and 2.

This study was deemed to not present a risk to patient safety or patient data protection by the trust’s chief clinical information officer. As this study formed part of a local service improvement project, no further formal ethics review was deemed necessary in keeping with appropriate guidelines [36].

### Data Analysis

The criteria in a completed questionnaire that were deemed to indicate the need for an FTF review were defined as the presence of any of the following: any grade 2 or higher response to a CTCAE-based question, having any symptom assessed with a binary question, or a PS in the range of 3-4. Using these criteria, the concordance between paired patient and clinician questionnaires for containing an FTF indicator was analyzed; the specific FTF indicator did not need to match in the paired questionnaires. Concordance was assessed by cross-tabulating the presence of an FTF indicator in paired patient- and clinician-completed questionnaires (Table 2).

**Table 2:** Cross tabulation of patient- and clinician-completed patient-reported outcome measure questionnaires by the presence of a face-to-face indicator.

Presence of an FTF^a^ indicator in patient-completed PROM^b^	Presence of an FTF indicator in clinician-completed PROM	
	Yes	No
Yes	True positive	False positive
No	False negative	True negative

^a^FTF: face-to-face.

^b^PROM: patient-reported outcome measure.

As the current standard of care comprises clinician interpretation of patient symptoms, the clinician-completed PROM represented the *standard*, and the patient-completed PROM represented the *test variable*. Sensitivity was calculated as true positive/(true positive+false negative) and specificity as true negative/(true negative+false positive). A similar method of cross-tabulation was performed to assess the presence of any severe binary symptoms or CTCAE grade 3 or higher symptoms in paired patient and clinician questionnaires.

The concordance of grading of common individual symptoms between paired patient and clinician questionnaires was assessed. Concordance was analyzed using descriptive statistics without the use of the Cohen κ statistic, as it was deemed to be the most accurate technique considering the predicted asymmetrical scoring differences in the ordinal data in line with recommendations from similarly conducted studies [25,27]. The Likert scale data from patient and staff feedback surveys were analyzed using descriptive statistics.

## Results

In total, 90 patients participated in the study across 10 different oncology treatment pathways, as shown in Table 1. The concordance between paired patient and clinician questionnaires for the presence of an indicator for FTF review is shown in Table 3.

**Table 3:** Concordance between paired patient-reported outcome measure questionnaires in highlighting the need for face-to-face review (N=90).

Presence of an FTF^a^ indicator in patient-completed PROM^b^ (n)	Presence of an FTF indicator in clinician-completed PROM (n)	
	Yes (n=47)	No (n=43)
Yes (61)	44	17
No (29)	3	26

^a^FTF: face-to-face.

^b^PROM: patient-reported outcome measure.

Thus, the sensitivity of the patient-completed questionnaires in correctly highlighting the need for FTF review was 94% (44/47) and specificity was 60% (26/43). False-negative patient-completed PROMs (ie, a patient questionnaire not indicating the need for FTF review but the clinician questionnaire indicating so) was 3% (3/90) of the total. Further analysis showed that these were all for symptoms that the clinician determined were of moderate severity (grade 2). Therefore, acknowledging these false negatives, 97% (87/90) of patient questionnaires flagged in a clinically appropriate manner.

All questionnaires were completed by participants in their entirety except for PS data being unavailable for 5 patients; 4 out of 5 of these patients had patient-completed questionnaires that already contained indicators for FTF review, with the remaining patient having corresponding patient- and clinician-completed questionnaires displaying no significant symptoms. Hence, the missing PS data were not considered likely to affect the above analysis.

Furthermore, 29% (26/90) of the paired questionnaires were concordant for the absence of any FTF indicators. This figure equates to the percentage of patients who were correctly identified by questionnaires not to need an FTF review and, therefore, the potential for FTF appointment reduction. Stratification by treatment pathway demonstrated that this percentage of potential FTF reduction by questionnaire varied considerably across the pathways from 0% (0/10) in patients receiving head and neck radical RT and 0% (0/5) in those receiving prostate chemotherapy to up to 70% (7/10) in those receiving single-agent immunotherapy, as shown in Table 4.

**Table 4:** Concordant questionnaires that contained no indicators for face-to-face review stratified by treatment pathway (N=90).

Treatment pathway (number of patients)	Concordant questionnaires per pathway without indicators for FTF^a^ review, n (%)
Immunotherapy (n=10)	7 (70)
Lung SABR^b^ (n=6)	3 (50)
Abiraterone and enzalutamide (n=10)	4 (40)
Pazopanib (n=10)	4 (40)
Imatinib (n=8)	3 (38)
Breast RT^c^ (n=10)	3 (30)
Prostate RT (n=10)	1 (10)
Colorectal chemotherapy (n=11)	1 (9)
Head and neck RT (n=10)	0 (0)
Prostate chemotherapy (n=5)	0 (0)

^a^FTF: face-to-face.

^b^SABR: stereotactic ablative radiotherapy.

^c^RT: radiotherapy.

Regarding concordance between paired patient and clinician questionnaires for the presence of a severe or grade 3+ symptom or higher (Table 5), the sensitivity of patient questionnaires was 100% (6/6) and specificity was 87% (73/84).

**Figure 2:**
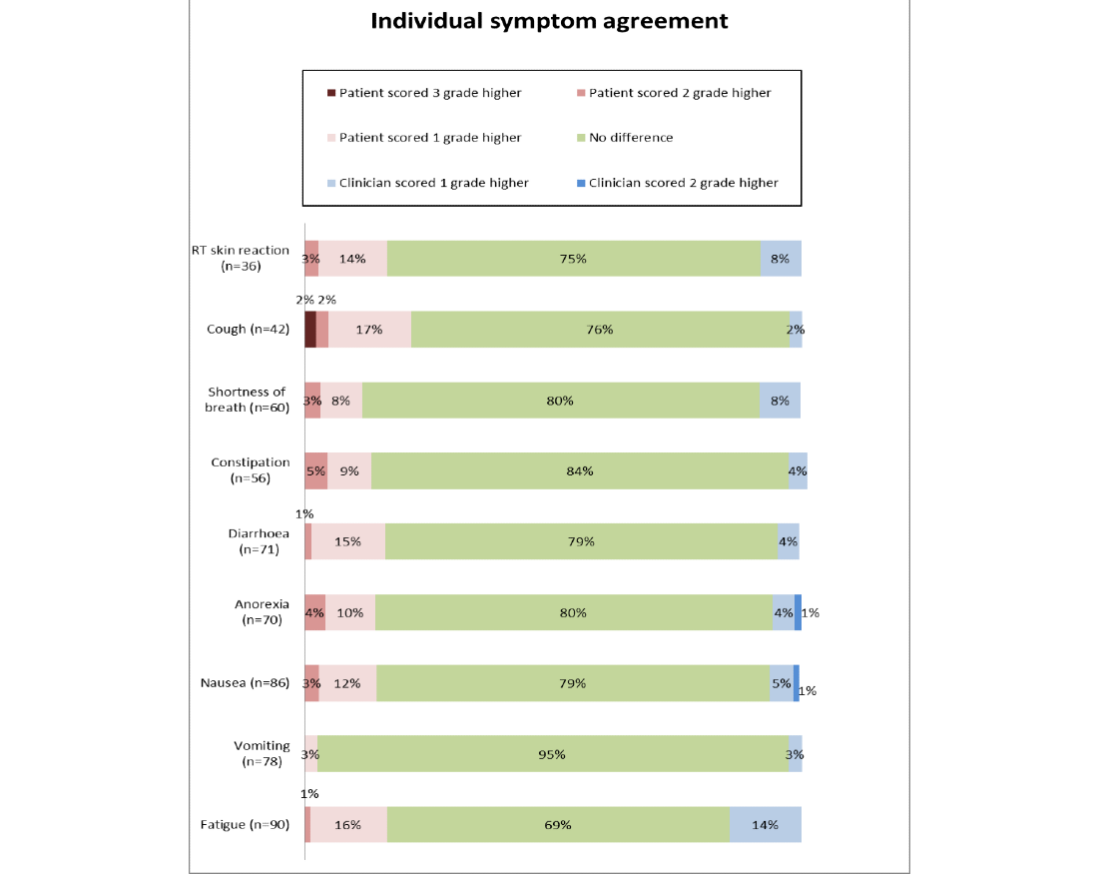
Agreement of common individual symptoms between patients and clinicians. RT: radiotherapy.

For frequently appearing symptoms in the different treatment-specific questionnaires (fatigue, vomiting, nausea, anorexia, diarrhea, constipation, shortness of breath, cough, and RT skin reaction), the exact agreement between patients and clinicians ranged from 69% (62/90) agreement for fatigue to 95% (74/78) for vomiting (Figure 2 and Multimedia Appendix 3). When there were individual symptom discrepancies between patients and clinicians, they were mostly within 1 grading point, and patients were more likely to assign greater severity to symptoms.

**Table 5:** The concordance between paired questionnaires for the presence of a severe symptom (N=90).

Presence of a severe or grade 3+ symptom in patient-completed PROM^a^ (n)	Presence of a severe or grade 3+ symptom in clinician-completed PROM (n)	
	Yes (n=6)	No (n=84)

Yes (17)	6	11
No (73)	0	73

^a^PROM: patient-reported outcome measure.

Of the 90 patients, 77 (86%) completed the feedback form. On a 10-point Likert scale, the mean patient approval score was 9.3 for usability, 9.1 for questionnaire thoroughness, and 8.8 for acceptance of questionnaires to supplement outpatient care. There were missing data for 3 patients who did not provide an answer for the latter question.

In addition, 48% (10/21) of clinicians completed a feedback form. On a 10-point Likert scale, the mean clinician approval score was 9.4 for usability, 9.8 for questionnaire thoroughness, and 9.6 for future use of the questionnaires to supplement outpatient care.

## Discussion

### Principal Findings

By comparing patient and clinician questionnaires, this study has shown that acute toxicity questionnaires based on patient-modified CTCAE can act as a triage tool to help highlight the need for FTF review in oncology *treatment* clinics. Patient questionnaires successfully detected all patients with severe symptoms. Our results indicate that the use of patient questionnaires to enable remote monitoring in certain treatment pathways could significantly reduce the need for FTF outpatient reviews. Patients and staff provided positive feedback on questionnaire usability and content and accepted its use to assist symptom monitoring. Our study thus contributes to the existing literature regarding the use of PROMs in routine outpatient settings [37-39], particularly the way that PROMs can usefully aid clinical decision-making and guide the need for FTF review in *on treatment* oncology clinics.

For common individual graded symptoms, the agreement between patients and clinicians was good; when there were differences, they were usually small, with patients more likely to indicate greater severity than clinicians, comparable with previous studies [25,27]. These individual differences rarely affected those patients who needed FTF review, with our results showing high sensitivity of our questionnaires, incorporating the presence of our predefined FTF indicators, to detect patients who needed FTF review and patients with potentially severe symptoms. This suggests there would be a low risk that patients who would potentially need clinical intervention would be missed. The tendency for some patients to rate symptoms more severely than clinicians explains the lower specificity of the questionnaires to determine the need for FTF review.

This study confirms the potential benefit of PROM questionnaires in acting as a triage tool for determining FTF review. Our results indicate that a significant proportion of participating patients (26/90, 29%) were correctly determined not to need an FTF appointment from their questionnaire results alone. There was a further proportion of patients, comprising 19% (17/90) of the cohort, in which the patient-completed questionnaires indicated a need for FTF review, but the corresponding clinician questionnaires suggested that this was not needed. This suggests that subsequent review of patients through a telephone or video consultation could be beneficial as a method to increase the specificity of patient questionnaires.

The study has also highlighted that the use of PROM questionnaires for the purpose of FTF reduction could be especially advantageous in certain follow-up treatment pathways. Of note, a large proportion of the pathways that seem particularly suitable for remote monitoring based on our results are the newer oncological systemic treatments, such as immunotherapy and tyrosine kinase inhibitor treatments. Patients can be on these treatments for many months and potentially years unlike traditional chemotherapy drugs where the course of treatment is usually a few months. Therefore, the benefits of appropriate FTF reduction to various stakeholders would be particularly discernible for patients with reduced hospital visits, leading to decreased burden on their time and finances, for clinicians with more productive use of their time, and for managers with more effective clinic use [9]. Moreover, PROMs have been shown to have broader clinical benefits for patients and clinicians [11], suggesting that more widespread use of digital PROMs would have additional health care benefits outside the primary scope of our study.

Technological solutions are being espoused to help with outpatient working [7,9], and our study demonstrates both patient and staff acceptance of our particular digital strategy. This helps justify that such an approach would work if it were to be implemented into routine oncological practice with both strong patient and staff willingness to drive its success. Digital PROMs are only one of the many technological tools that can help make oncology work more efficiently. Video consultations to enable remote review of patients have been shown to be safe and effective when used appropriately [40], and their use has expanded rapidly in response to the COVID-19 pandemic [41]. Other technological solutions that seek to improve the efficiency of a number of aspects of oncology work include artificial intelligence applied to radiomics, such as breast screening interpretation [42], and streamlining RT workflows, such as through auto-contouring during RT outlining and voxel-based dose prediction approaches to refine the treatment planning process [43]. Thus, digital technology, including electronic PROMs, looks set to have a significant impact on oncology practice.

### Limitations

The questionnaires in this study were largely based on CTCAE, which has the limitation of not being formally validated [27]. However, they form the standard for adverse event reporting in oncology [22], and in line with previous studies [27,29], modification of terminology into patient-understandable language enables patient reporting of symptom severity while mapping onto an established grading system that is well known and widely used by clinicians. Furthermore, the UK Oncology Nursing Society triage tool was used for the emergency assessment of chemotherapy toxicity in our study center [44] and the UK Oncology Nursing Society tool is based on CTCAE criteria; thus, this was felt to additionally aid acceptance of the digital PROMs used by clinicians in this study. Currently, the National Cancer Institute’s patient-reported outcome tool PRO-CTCAE does not map exactly onto the recognized CTCAE grading system; therefore, it would be difficult to use it as a remote monitoring tool to determine the need for FTF assessment. Furthermore, CTCAE has been applied to other specialties outside of oncology, such as in trials pertaining to hypertension and HIV [45], making it generalizable to other medical specialties.

In this study, clinicians were asked to complete the PROM questionnaires based on information gathered from routine FTF appointments. The questions asked in these FTF consultations were up to the clinician’s discretion as per their routine practice. Therefore, there is the possibility that clinicians may have completed PROM questionnaires with insufficient information. This limitation reflects the standard clinical practice for *on treatment* reviews, which would have a less *systematic* approach than a PROM questionnaire.

Potential FTF reduction using digital questionnaires was estimated through the absence of predefined indicators for FTF review. The authors recognize that patients may want to see their medical team in an FTF appointment for reasons other than these indicators. Actual FTF reduction will, therefore, likely be lower in practice, but these figures demonstrate the large opportunity for follow-up reduction if an appointment is deemed unnecessary from both the patient and clinician perspectives. If such questionnaires are to be applied to routine care, questionnaires should be designed to allow patients to explicitly state their request for an FTF review to enable a patient-centered approach to care.

It can be stated that our study is limited by the fact that statistical analyses, such as Cohen κ statistics, were not used to formally assess agreement between patients and clinicians. However, previous studies have criticized Cohen κ statistics in this setting because of the asymmetry across the scoring differences and have advocated descriptive statistics, as used in this study, as being sufficient for determining interrater concordance in this particular situation.

Although the patient feedback form response rate was high at 86% (77/90), it was recognized that the staff feedback form response rate was significantly lower at 48% (10/21). This is partly explained as patients were asked to complete this directly after their FTF appointment following an in-person request from a member of the project team, whereas clinicians were asked to do this via email after the study was completed. Hence, nonresponse bias may affect the strength of the conclusions that can be drawn from the staff feedback data.

### Future Work

Moving forward, we have organized patient focus groups to provide detailed qualitative feedback about patient understanding and acceptance of the designed questionnaires. These will occur before a planned pilot study to use *remotely completed* digital PROMs in selected oncology treatment pathways to assess their ability to reduce the need for FTF care. We are also considering remote monitoring for patients who have completed their cancer treatment via PROMs as part of their long-term follow-up. These PROM questionnaires would target symptoms suggestive of recurrence as well as the consequences of their cancer treatment.

### Conclusions

This study demonstrates the potential efficacy and utility of PROM questionnaires to facilitate remote monitoring of patients undergoing oncology treatments to reduce the need for FTF care. They have a high approval rating from both patients and clinicians. Significantly, they appeared to correctly identify patients with severe adverse treatment effects. From our data, a treatment strategy using digital PROMs in our oncology center alone, which has approximately 30,000 follow-up attendances per year, could safely reduce the need for thousands of FTF appointments. The use of remote monitoring via PROMs could lead to a more patient-centered model of care with a reduced need for hospital visits with resultant benefits to patients, clinicians, and the wider health system.

## Appendix

Multimedia Appendix 1 Patient feedback questions.

Multimedia Appendix 2 Clinician feedback questions.

Multimedia Appendix 3 Raw data set for individual common symptom agreement between patients and clinicians.

## Abbreviations

CTCAE: Common Terminology Criteria for Adverse Events
FTF: face-to-face
NHS: National Health Service
PRO-CTCAE: patient-reported outcomes Common Terminology Criteria for Adverse Events
PROM: patient-reported outcome measure
PS: performance status
RT: radiotherapy
