# Supplementary material for: Digital Patient-Reported Outcome Measures for Monitoring of Patients on Cancer Treatment: Cross-sectional Questionnaire Study
Source: JMIR Form Res. 2021 Aug 13;5(8):e18502. doi: 10.2196/18502 (PMC8398740; doi:10.2196/18502)
Supplement: Multimedia Appendix 1 [file formative_v5i8e18502_app1.docx]

**How confident were you with reading and answering the questions on the tablet?**

☹ 1 2 3 4 5 6 7 8 9 10 ☺

**Did the questions cover everything you would normally discuss at clinic?**

☹ 1 2 3 4 5 6 7 8 9 10 ☺

**Would you be happy to use these digital questionnaires routinely to supplement your care?**

☹ 1 2 3 4 5 6 7 8 9 10 ☺
