# Supplementary material for: Digital Patient-Reported Outcome Measures for Monitoring of Patients on Cancer Treatment: Cross-sectional Questionnaire Study
Source: JMIR Form Res. 2021 Aug 13;5(8):e18502. doi: 10.2196/18502 (PMC8398740; doi:10.2196/18502)
Supplement: Multimedia Appendix 2 [file formative_v5i8e18502_app2.docx]

How user friendly did you find the software?

☹ 1 2 3 4 5 6 7 8 9 10 ☺

Did the Questionnaire include all of the relevant acute toxicity questions you would want to ask?

☹ 1 2 3 4 5 6 7 8 9 10 ☺

Would you be happy to use these questionnaires as a means of remote patient review and monitoring?

☹ 1 2 3 4 5 6 7 8 9 10 ☺
